# Metabolic Profiling of the Diabetic Heart: Toward a Richer Picture

**DOI:** 10.3389/fphys.2019.00639

**Published:** 2019-05-31

**Authors:** Alice P. Sowton, Julian L. Griffin, Andrew J. Murray

**Affiliations:** ^1^Department of Physiology, Development and Neuroscience, University of Cambridge, Cambridge, United Kingdom; ^2^Department of Biochemistry and Systems Biology Centre, University of Cambridge, Cambridge, United Kingdom

**Keywords:** diabetic cardiomyopathy, heart failure, metabolomics, lipidomics, mitochondria, animal models

## Abstract

The increasing global prevalence of diabetes has been accompanied by a rise in diabetes-related conditions. This includes diabetic cardiomyopathy (DbCM), a progressive form of heart disease that occurs with both insulin-dependent (type-1) and insulin-independent (type-2) diabetes and arises in the absence of hypertension or coronary artery disease. Over time, DbCM can develop into overt heart failure. Like other forms of cardiomyopathy, DbCM is accompanied by alterations in metabolism which could lead to further progression of the pathology, with metabolic derangement postulated to precede functional changes in the diabetic heart. Moreover in the case of type-2 diabetes, underlying insulin resistance is likely to prevent the canonical substrate switch of the failing heart away from fatty acid oxidation toward increased use of glycolysis. Analytical chemistry techniques, collectively known as metabolomics, are useful tools for investigating the condition. In this article, we provide a comprehensive review of those studies that have employed metabolomic techniques, namely chromatography, mass spectrometry and nuclear magnetic resonance spectroscopy, to profile metabolic remodeling in the diabetic heart of human patients and animal models. These studies collectively demonstrate that glycolysis and glucose oxidation are suppressed in the diabetic myocardium and highlight a complex picture regarding lipid metabolism. The diabetic heart typically shows an increased reliance on fatty acid oxidation, yet triacylglycerols and other lipids accumulate in the diabetic myocardium indicating probable lipotoxicity. The application of lipidomic techniques to the diabetic heart has identified specific lipid species that become enriched and which may in turn act as plasma-borne biomarkers for the condition. Metabolomics is proving to be a powerful approach, allowing a much richer analysis of the metabolic alterations that occur in the diabetic heart. Careful physiological interpretation of metabolomic results will now be key in order to establish which aspects of the metabolic derangement are causal to the progression of DbCM and might form the basis for novel therapeutic intervention.

## Introduction

Expansion of the global population and an increase in age-standardized prevalence (Palermo et al., 2014) have together contributed to a rapid escalation in the worldwide incidence of diabetes mellitus (NCD Risk Factor Collaboration (NCD-RisC), 2016). Whilst diabetes is associated with a wide variety of clinical complications, cardiovascular diseases account for approximately 65% of diabetes-related mortality (Pappachan et al., 2013). CAD and hypertension are both highly prevalent in the diabetic population, however the risk of heart failure is elevated in patients with diabetes even when CAD and hypertension are taken into account (Hölscher et al., 2016). This increased susceptibility to failure is attributed to a collection of metabolic, functional, and morphological derangements to the myocardium, collectively termed DbCM. Clinically, DbCM is defined as the presence of myocardial dysfunction in a diabetic patient in the absence of classical cardiovascular risk factors, such as CAD, hypertension, or valve disease (Jia et al., 2018). The very existence of DbCM has been considered controversial, although it is now generally accepted as a distinct form of heart disease demonstrable in both animal models and human patient cohorts (Ernande and Derumeaux, 2012). Prolonged hyperglycemia, accompanied by systemic insulin resistance in the case of type-2 diabetes (T2DM), is considered central to the etiology of DbCM (Bell, 2003). It is a progressive condition that initially presents as subclinical ventricular hypertrophy and fibrosis, but can develop into overt heart failure with systolic dysfunction (Jia et al., 2018).

Heart failure is classically defined as an inability of the heart to deliver sufficient blood to the tissues of the body to meet metabolic demands (Braunwald, 1978). It can arise due to a number of underlying causes and is a multifactorial condition, but alterations in cardiac metabolism and energetics are now considered to be important features of the underlying pathology (Neubauer, 2007). The healthy heart is metabolically flexible, predominantly employing FA oxidation to meet ATP requirements, but with a further significant contribution from glucose oxidation depending upon circumstances, and smaller contributions from the oxidation of lactate, ketone bodies, and BCAAs (Bing et al., 1954). It is commonly reported that the failing heart undergoes a substrate ‘switch’ with the proportion of ATP derived from glycolysis exceeding that from FA oxidation (Neubauer, 2007). This may represent an overall impairment in oxidative capacity, since several studies have reported an impairment in glucose oxidation in the failing heart (Zhabyeyev et al., 2013; Seymour et al., 2015; Diakos et al., 2016; Kaimoto et al., 2017), with a decreased mitochondrial OXPHOS capacity being associated with a diminished energetic reserve in the myocardium (Stanley et al., 2005). Furthermore, the failing heart becomes insulin resistant (Swan et al., 1994), eventually limiting the capacity for glucose catabolism to meet energetic demands, with ketone body oxidation possibly increasing in later stages of the condition (Aubert et al., 2016; Bedi et al., 2016).

Diabetic cardiomyopathy, however, is considered to differ from other causes of heart failure. Metabolic disturbances precede the first signs of mechanical failure in the diabetic myocardium and are considered likely to be causative (Jia et al., 2018). Moreover, in contrast with animal models of, e.g., pressure-overload or ischemic heart failure (Rosca and Hoppel, 2010), mitochondrial content has been reported to be increased in the hearts of diabetic rodents, whilst proportionally, FA oxidation increases and glucose oxidation is depressed (Bugger and Abel, 2010). Whilst this metabolic profile would appear to differ from that of the failing non-diabetic heart, what unifies the two pathologies is a loss in metabolic flexibility, potentially impairing the capacity of the heart to utilize the most appropriate substrate depending on circumstance. Whilst metabolic and energetic derangements are considered to be core to the etiology of DbCM and the progression to failure, the time-course of metabolic alterations with respect to functional and morphological changes, and in conjunction with eventual failure of the myocardium, remains unclear.

The growing field of metabolomics employs a collection of analytical techniques which have the potential to elucidate this progression of metabolic disturbances in the diabetic heart and may additionally highlight novel plasma-borne biomarkers. In this review, we first give a brief overview of some of the most commonly used metabolomic techniques and models, before considering in detail those studies that have applied these techniques to investigate metabolic changes in the diabetic heart. In particular, we draw attention to the growing field of lipidomics and how alterations to plasma lipid species could serve as putative biomarkers for DbCM. Finally, we highlight how existing and emerging metabolomic technologies could be used to address some of the remaining questions concerning cardiac metabolism in diabetes.

## An Overview of Metabolomics and Metabolomic Methods

The comprehensive study of all metabolites in a biological system is referred to as metabolomics (Dunn et al., 2011). In comparison with proteins and nucleic acids, metabolites have a relatively low molecular weight (<1500 Da), exist across a wide range of concentrations and have extremely diverse chemical properties (Dunn et al., 2011). This can make the detection, identification and (semi-) quantification of metabolites a technical challenge. Nevertheless, the application of analytical chemistry techniques such as NMR spectroscopy, MS and chromatography, coupled to the production and expansion of data libraries such as the Human Metabolome Database (Wishart et al., 2007), has led to the development of both unbiased and targeted assays for high-throughput metabolic profiling and analysis (O’Brien et al., 2015).

### Nuclear Magnetic Resonance Spectroscopy

Nuclear magnetic resonance utilizes a quantum property of certain atomic nuclei, known as spin, to visualize the structure of a chemical species. When exposed to a magnetic field, these nuclei assume different energy levels which can be detected using radio waves (Griffin et al., 2011). The chemical environment of a particular nucleus affects the resonant frequency at which that nucleus will be detected, a phenomenon known as the chemical shift, and as such structural information can be determined from NMR spectra (Griffin et al., 2011). Atoms such as ^1^H, ^13^C, and ^31^P have nuclei with a quantum spin number of ½, and therefore have two energy levels which can be detected by NMR spectroscopy. Helpfully, these nuclei are also ubiquitous across metabolic species in biological systems, making NMR a highly valuable tool in metabolomic studies (Griffin et al., 2011). ^1^H-NMR is particularly useful in metabolomic analysis owing to its greater relative sensitivity, which arises from the higher gyromagnetic ratio of ^1^H in comparison with other nuclei (Derome, 1987).

Nuclear magnetic resonance is non-destructive, allowing multiple analyses to be carried out on a single sample (Dunn et al., 2011). This has also permitted application of the technique to living systems, allowing the *in vivo* measurement of metabolites in MRS studies (see for example, Szczepaniak et al., 2003; Reingold et al., 2005; Perseghin et al., 2007; Bilet et al., 2011). Although only a small number of metabolites can be measured *in vivo*, these include ATP and PCr using ^31^P-MRS, allowing tissue energetics to be investigated. Moreover, biopsy samples are not required and the technique does not use ionizing radiation, thus MRS has increased the potential for recruiting patient cohorts of a meaningful size for studies of metabolism (e.g., Neubauer et al., 1997).

The structural information that can be determined from NMR spectra can allow the identification of metabolites in an unbiased manner (Dunn et al., 2011). A major disadvantage of NMR, however, is its low sensitivity especially in relation to other metabolomic techniques, and it is therefore limited to the detection of metabolites present at high concentrations *in vivo* (Dunn et al., 2011). One method that has increased the sensitivity of such measurements is hyperpolarized NMR (Schroeder et al., 2008, 2009, 2011). Hyperpolarization improves sensitivity by enhancing the polarization of the nucleus of interest, thereby increasing the signal that can be detected by an NMR/MRS scanner (Golman et al., 2008). Through the introduction of a specific metabolite labeled with hyperpolarized ^13^C, for example, the enzymatic conversion through metabolic pathways, such as the TCA cycle, can be observed in real time with a resolution as low as 1 s (Schroeder et al., 2009). However, a major limitation of hyperpolarized NMR is that only a small number of metabolites (e.g., [1-^13^C]-pyruvate) can be usefully studied, and this depends on both physicochemical properties (e.g., relaxation, polarization) and biological properties (e.g., safety, pharmacokinetics) of the molecule (Miloushev et al., 2016). Hyperpolarized NMR spectroscopy is therefore highly useful when measuring the *in vivo* kinetics of a particular enzyme of interest, such as PDH (Schroeder et al., 2008; Atherton et al., 2011), but is not suitable for identifying more global metabolic changes.

### Mass Spectrometry

Mass spectrometry initially requires the ionization of analytes in a sample of interest, before the separation and detection of individual ions on the basis of their mass-to-charge (*m/z*) ratio (Dunn et al., 2011). Determination of the specific analyte, or metabolite in the case of metabolomics, is achieved through comparison of spectra with pure chemical standards or publicly-available data repositories. MS is a highly sensitive technique, and is able to detect and identify significantly more metabolites than is possible with NMR (Griffin et al., 2011). It can, however, be difficult to distinguish between different isomers of molecules, or between non-isomeric species of the same molecular weight (Griffin et al., 2011).

Mass spectrometry is often coupled to chromatographic separation, usually either GC or LC, which can greatly expand the number of metabolites that can be detected (Dunn et al., 2011). Samples can, however, also be directly introduced to mass spectrometers, often in an automated manner, increasing the throughput of the analysis (Dunn et al., 2011). This method of direct-infusion mass spectrometry (DIMS) requires high resolution instrumentation to ensure adequate discrimination of metabolite species (Griffin et al., 2011). DIMS is often used in lipid analysis, where it is sometimes referred to as shotgun lipidomics (Griffin et al., 2011), although it can also be used to profile aqueous metabolites.

### Chromatography

Chromatography refers to the separation of different molecular species in a mixture, such as a biofluid or tissue extract. A column is utilized, through which different analytes pass at different rates depending on their chemical properties and that of the column used. The time taken for a particular metabolite to elute from the chromatographic column is referred to as the retention time, and this can be used for molecular identification.

Two chromatographic techniques are commonly employed in metabolomic studies: GC and LC, which differ in the phase in which metabolites are separated. GC uses a long column to separate metabolites in the gaseous phase. This can be beneficial when coupled to MS, since metabolites are already volatile when entering the mass spectrometer (Griffin et al., 2011). LC separates metabolites dissolved in a liquid by partitioning them between the liquid and solid phase. However, because metabolites are in the liquid phase they cannot be directly introduced to a mass spectrometer following elution from the column. To overcome this, ESI is often used, whereby the metabolite-containing liquid is aerosolized, forming charged particles that can then enter the mass spectrometer (Griffin et al., 2011). GC is considered more robust than LC in terms of reproducibility, since retention time shift during any given analytical run tends to be more modest (Griffin et al., 2011). However, as many metabolites are not volatile, samples often have to be derivatized before GC analysis, which is often not the case in LC and more metabolites can therefore be analyzed by LC–MS compared with GC–MS (Griffin et al., 2011).

Although chromatography is usually coupled to MS, other techniques for identifying metabolites following chromatographic separation can also be employed. Examples of alternative detectors include FIDs and TCDs, which can be used to identify analytes as they are eluted from GC columns (Zuo et al., 2013).

### Targeted Analysis Versus Open-Profiling

Metabolomic studies are often subdivided into open-profiling and targeted analyses. Open-profiling involves detecting and measuring all (or at least as many as possible) of the metabolites in a sample, and is therefore sometimes referred to as unbiased metabolomics (Dunn et al., 2011). Such studies are typically hypothesis-generating and are useful, for instance, in the discovery of biomarkers in particular disease states, as well as for the discovery of novel metabolites associated with a particular process (Griffin et al., 2011). Targeted analysis, however, involves the measurement of a limited number of pre-defined metabolites, and is therefore typically employed in hypothesis-testing experiments (Griffin et al., 2011). Targeted analysis is often more sensitive than open-profiling, and can be used to provide absolute quantification of metabolites of interest (Dunn et al., 2011). In the particular case of DbCM, targeted analysis has generally been used to investigate changes in energetic status or substrate preference in the diabetic heart (see sections “High-Energy Phosphate Metabolism in the Diabetic Heart” and “Substrate Preference of the Diabetic Heart: Glucose and Fatty Acid Metabolism”), whilst more open-profiling lipidomic techniques are being employed to investigate changes to individual lipid species that may serve as putative biomarkers for the condition (see section “Lipidomics: A Tool for Biomarker Discovery in DbCM”).

## Animal Models of Diabetes, Obesity, and the Metabolic Syndrome

A number of animal models of diabetes, obesity and the metabolic syndrome [a pre-diabetic state characterized by obesity, hypertension, hyperglycemia, hyperinsulinemia, and dyslipidemia (Dandona et al., 2005)] have been developed and characterized, and these have been comprehensively reviewed in a number of previous publications (Rees and Alcolado, 2005; Bugger and Abel, 2009; Lutz and Woods, 2012; Wang et al., 2014; Wong et al., 2016). Many of these models have been vital in the research of DbCM, including in metabolomic studies, and some of the key models will be briefly introduced here.

Rodents are the most commonly used animal models of diabetes, and, broadly speaking, they can be divided into genetic and inducible models. The most common genetic models are summarized below (Table 1). A number of these models arose from mutations in the genes encoding either the satiety hormone leptin [e.g., the *ob/ob* mouse (Friedman et al., 1991; Zhang et al., 1994)] or that of its receptor [e.g., the *db/db* mouse (Hummel et al., 1966; Chen et al., 1996)]. The Zucker fatty rat, which is obese and shows features of the metabolic syndrome (Zucker and Zucker, 1961; Zucker and Antoniades, 1972), also resulted from a mutation in the leptin receptor gene, whilst selective breeding of Zucker fatty rats gave rise to the more severe phenotype of the ZDF rat (Shiota and Printz, 2012; Lehnen et al., 2013). In all cases, these loss-of-function mutations result in hyperphagia, with the rodents experiencing chronic over-nutrition which rapidly develops into obesity and hyperinsulinemia, and can eventually lead to β-cell dysfunction and a severe diabetic phenotype similar to the clinical manifestation of T2DM (Wang et al., 2014). In contrast, the Akita mouse represents a genetic model of T1DM, brought about via a missense mutation in the gene encoding insulin (Yoshioka et al., 1997; Wang et al., 1999). Another common genetic model of T1DM is the non-obese diabetic (NOD) mouse, a polygenic model which develops the disease through autoimmune destruction of the pancreatic β-cells (Wicker et al., 1987; Serreze and Leiter, 1994).

**Table 1:** Common genetic rodent models of diabetes, obesity, and the metabolic syndrome.

Model	Species	Gene mutation	Condition Modeled
Akita	Mouse	Insulin missense	T1DM (Yoshioka et al., 1997; Wang et al., 1999)
NOD	Mouse	Polygenic	T1DM (Wicker et al., 1987; Serreze and Leiter, 1994)
*db/db*	Mouse	Leptin receptor, splice variant	T2DM (Hummel et al., 1966; Chen et al., 1996)
*ob/ob*	Mouse	Leptin, nonsense	Obesity, features of T2DM/MetS (Friedman et al., 1991; Zhang et al., 1994)
Zucker Fatty	Rat	Leptin receptor, missense	Obesity, features of MetS (Zucker and Zucker, 1961; Zucker and Antoniades, 1972)
ZDF	Rat	Leptin receptor, missense	T2DM (Shiota and Printz, 2012; Lehnen et al., 2013)

*T1DM, type-1 diabetes; T2DM, type-2 diabetes; MetS, metabolic syndrome; NOD, non-obese diabetic; ZDF, Zucker diabetic fatty.*

**Table 2:** Results from ^31^P-MRS studies in diabetic rodents and patients.

Species	Measurement	Model/population	PCr/ATP compared with controls	References
Mouse	*In vivo*	HFD	–	Abdurrachim et al., 2014
Mouse	*In vivo*	*db/db*	↓	Abdurrachim et al., 2017
Mouse	*In vivo*	*db/db* + TAC	–	Abdurrachim et al., 2017
Rat	*In vivo*	STZ	–	Bollano et al., 2007
Rat	*Ex vivo*	STZ	↓	Liang et al., 2015
Rat	*Ex vivo*	STZ	-^∗^	Spindler et al., 1999
Human	*In vivo*	Newly diagnosed T2DM, well-controlled	↓	Diamant et al., 2003
Human	*In vivo*	T2DM on oral antidiabetic therapy	↓	Levelt et al., 2016b
Human	*In vivo*	Normotensive T2DM subjects	↓	Levelt et al., 2016a
Human	*In vivo*	Male T2DM	–	Rijzewijk et al., 2009
Human	*In vivo*	T2DM, 18–75 years old, no evidence of cardiac ischemia	↓	Scheuermann-Freestone et al., 2003

*–No difference in PCr/ATP between diabetic and control groups. ↓ PCr/ATP is lower in diabetic subjects compared with controls. ^∗^Inferred from measured levels of PCr and ATP. HFD, high fat diet; TAC, thoracic aortic constriction; STZ, streptozotocin; T2DM, type-2 diabetes.*

Whilst the models listed in Table 1 arose due to spontaneous mutations that occurred in laboratory rodent colonies, modern genetic engineering techniques have enabled generation of mice with specific desired mutations. Examples of such animals that have been used in metabolomic studies of DbCM include the βV59M mouse, a model of human neonatal diabetes that can be rapidly induced and reversed, which has a mutation in the pancreatic β-cell ATP-sensitive K^+^ channel (Girard et al., 2009; Rohm et al., 2018), and the glycerol-3-phosphate acyltransferase 1 knockout (*Gpat1^−/−^*) mouse which exhibits a diabetic phenotype owing to alterations to cardiac glycerol-phosphate metabolism (Lewin et al., 2008).

As an alternative to genetic models, diabetes can be induced through administration of a diabetogenic toxin or diet. The antibiotic, STZ, or alloxan (a toxic glucose analog) are commonly used to induce diabetes in rats or mice through the destruction of pancreatic β-cells (Shaw Dunn et al., 1943; Rossini et al., 1977). Such compounds can be administered intravenously (*i.v.*) or intraperitoneally (*i.p.*) (Rossini et al., 1977), and result in the development of a severe T1DM phenotype (Wu and Yan, 2015).

High fat diets are often used to induce obesity and the metabolic syndrome in rodent models (Buettner et al., 2007). Since insulin resistance and/or glucose intolerance are characteristics of metabolic syndrome, HFD-fed rodent models are commonly used for investigations into T2DM (Buettner et al., 2007), however overt T2DM does not always develop with HFD feeding. In order to expedite the development of T2DM, high-fat, high-sugar diets (with high fructose or sucrose content) can be used, which are especially diabetogenic on certain rodent backgrounds such as the C57BL/6J mouse (Surwit et al., 1988). Alternatively, high-fat feeding has been combined with a low-dose injection of STZ to generate a reproducible T2DM phenotype (Srinivasan et al., 2005). Such HFD models have been used to investigate alterations to cardiac metabolism in the diabetic heart by both metabolomic (e.g., Mansor et al., 2013; Abdurrachim et al., 2014; Le Page et al., 2015) and non-metabolomic methods, such as radio-labeled substrate utilization or measurements of mitochondrial respiratory capacity (e.g., Reid et al., 1970; Cole et al., 2011; Boardman et al., 2017).

## High-Energy Phosphate Metabolism in the Diabetic Heart

It has long been postulated that the failing heart is energy-starved, with the hypothesis first put forward in the 1930s (Herrmann and Decherd, 1939). The advent of MRS technology, and specifically ^31^P-MRS, has allowed measurements to be made of the myocardial PCr to ATP ratio (PCr/ATP) *in vivo* as an index of cardiac energy reserve. The creatine kinase system acts to temporally and spatially buffer ATP in the myocardium, transferring phosphate groups from ATP to creatine at the mitochondria and from PCr to ADP at the myofibrils (Wallimann et al., 2011). This acts to maintain ADP levels in the mitochondria to support OXPHOS and ATP levels at the myofibrils to support contraction, particularly under conditions of increased ATP demand (Neubauer, 2007). A fall in myocardial PCr/ATP can therefore indicate that ATP synthesis is not keeping pace with cellular energy demand.

PCr/ATP is decreased in failing human hearts, appearing to confirm the energy-starvation hypothesis, and this index was additionally found to predict mortality in patients with dilated cardiomyopathy (DCM) (Neubauer et al., 1997). ^31^P-MRS has also been employed to measure PCr/ATP in diabetic patients, as well as animal models, however evidence that the diabetic myocardium is energy-starved is a little less clear (Table 2). Whilst a number of studies in both rodents and humans have demonstrated a lower PCr/ATP in the diabetic myocardium (Diamant et al., 2003; Scheuermann-Freestone et al., 2003; Liang et al., 2015; Levelt et al., 2016a,b; Abdurrachim et al., 2017), others found no difference in myocardial energy reserve compared with controls (Spindler et al., 1999; Bollano et al., 2007; Rijzewijk et al., 2009; Abdurrachim et al., 2014).

These apparent discrepancies may be explained by the model or population studied. All human studies considered in Table 2 were carried out in T2DM cohorts, and here there is a general consensus that PCr/ATP is depressed in the myocardium (Diamant et al., 2003; Scheuermann-Freestone et al., 2003; Levelt et al., 2016a,b). The one human study that found no difference in PCr/ATP used a patient population that were transferred to glimepiride monotherapy 8-weeks prior to the measurements taking place (Rijzewijk et al., 2009). Glimepiride is a sulfonylurea-class anti-diabetic therapy that increases insulin secretion from pancreatic β-cells through blockade of the ATP-dependent potassium channels (Davis, 2004). Although glimepiride is believed to be pancreas-specific, it is possible that it may beneficially affect the cardiovascular system (Davis, 2004), either directly or indirectly via improvements in insulin secretion. Whilst the other human studies considered in Table 2 include patients taking sulfonylureas, they also included patients on other forms of oral anti-diabetic agents, such as metformin, and thus the anomalous results may reflect a specific effect of glimepiride.

**Table 3:** Changes in FA and carbohydrate metabolism in the diabetic heart as reported by metabolomic studies in comparison with non-diabetic controls.

Metabolic pathway				Metabolomic method	Experimental model	References
			
Glycolysis	Glucose oxidation	Pyruvate oxidation	Fatty acid oxidation			
	↓			NMR	STZ rat	Chatham and Forder, 1993
	↓		↓	NMR	STZ rat	Chatham and Forder, 1997
			↓	NMR	STZ rat	Chatham et al., 1999
↓	↓			NMR	ZDF rat	Chatham and Seymour, 2002
↓			↑	NMR	ZDF rat	Wang et al., 2005
		↓		^13^C MRS^∗^	STZ rat	Schroeder et al., 2008
		↓		^13^C MRS^∗^	STZ rat + HFD	Le Page et al., 2015
		↓		^13^C MRS^∗^	βV59M mice	Rohm et al., 2018

*Note that evidence from non-metabolomic studies more conclusively supports an increase in FA oxidation. ↓ decreased pathway flux in diabetes, ↑ increased pathway flux in diabetes. ^∗^ Indicates the use of hyperpolarized NMR spectroscopy. NMR, nuclear magnetic resonance; ^13^C MRS, carbon-13 magnetic resonance spectroscopy; STZ, streptozotocin; ZDF, Zucker diabetic fatty; HFD, high fat diet.*

Likewise, the different rodent models investigated reflect slightly different diabetic states. The finding of lower PCr/ATP in the *db/db* mouse (Abdurrachim et al., 2017) supports the human data suggesting myocardial energy depletion in T2DM. However, when exposed to pressure overload via TAC the cardiac energetics of *db/db* mice were normalized (Abdurrachim et al., 2017). It was proposed that this might be a result of restoration of cardiac metabolic substrate balance, whereby pressure-overload leads to increased glucose uptake and utilization thereby reducing the reliance on FA oxidation in the diabetic heart (Abdurrachim et al., 2017). This suggests the metabolic etiology of DbCM contrasts with that of pressure overload-induced heart failure. Despite also being a model of insulin resistance, the discrepancy between the unaltered PCr/ATP in HFD-fed mice (Abdurrachim et al., 2014) compared with overtly-diabetic *db/db* mice may reflect differences in the degree of the pathology induced. Indeed, although fasting plasma glucose levels were elevated in mice with HFD feeding, plasma insulin levels were not significantly raised (Abdurrachim et al., 2014), suggesting this model may reflect a pre-diabetic or glucose intolerant state, rather than overt T2DM.

The STZ rat differs from other models, in that it represents a form of T1DM. In the STZ rat, different results have been reported concerning PCr/ATP when measured across three studies, with one study showing worsened energetic reserve (Liang et al., 2015) and two suggesting no difference (Spindler et al., 1999; Bollano et al., 2007). These discrepancies may be explained by the different strains of rat used [Wistar (Spindler et al., 1999) vs. Sprague-Dawley (Bollano et al., 2007; Liang et al., 2015)], dose of STZ [65 mg kg^−1^ (Spindler et al., 1999; Bollano et al., 2007) vs. 70 mg kg^−1^ (Liang et al., 2015)], route of STZ injection [*i.p.* (Liang et al., 2015) vs. *i.v.* (Spindler et al., 1999; Bollano et al., 2007)], study length [4 weeks (Spindler et al., 1999) vs. 6 weeks (Spindler et al., 1999; Liang et al., 2015) vs. 12 weeks (Bollano et al., 2007)] or the age of the rats when diabetes was induced [5–6 weeks (Spindler et al., 1999) vs. 8 weeks (Liang et al., 2015), age not stated in Bollano et al. (2007)]. Such differences in study design are known to confound interpretation of results in studies utilizing STZ-induced diabetes (Deeds et al., 2011).

Although PCr/ATP is considered to be a good marker for cellular energy reserve, there are many factors which could also explain some of the different results reported in Table 2. For example myocardial PCr/ATP was found to become depleted in association with elevated plasma FA levels in healthy young men, either as a result of fasting and exhaustive exercise (Bilet et al., 2011) or a high-fat low-carbohydrate diet (Holloway et al., 2011). Meanwhile, weight-loss was found to improve myocardial PCr/ATP in obese patients, suggesting whole-body energetic status can impact myocardial PCr/ATP (Rider et al., 2013). This is supported by a study in healthy men, which found that PCr/ATP decreased with increased body mass index (Perseghin et al., 2007). A further study in a male T2DM cohort, however, found that two anti-glycemic agents, metformin and pioglitazone, did not affect cardiac high-energy phosphate levels (van der Meer et al., 2009).

Current evidence therefore supports the notion of energy depletion in the T2DM heart, and this may depend upon the severity of the condition. Whether myocardial energy depletion is a causal factor in the progressive development of DbCM, or simply a consequence of the metabolic derailment remains unknown.

## Substrate Preference of the Diabetic Heart: Glucose and Fatty Acid Metabolism

A classic metabolic signature of heart failure, occurring for example during the development of pressure overload hypertrophy, is a relative switch in substrate preference away from FA oxidation and toward glucose metabolism, particularly glycolysis (Neubauer, 2007). In the diabetic heart, evidence suggests that remodeling of substrate preference does occur, albeit in the opposite direction to that following pressure overload, however results from metabolomic studies are somewhat inconsistent (Table 3). The most common and consistent finding is that glucose metabolism, encompassing anaerobic glycolysis and glucose oxidation, is suppressed in the diabetic myocardium. This is in accordance with the pathology of diabetes; insulin resistance in T2DM and lack of insulin in T1DM result in impaired glucose uptake, whilst increased circulating FFA concentrations (in both T1DM and T2DM) suppress glucose oxidation through the action of the Randle cycle. This occurs through inhibition of glycolysis and pyruvate oxidation mediated by increased acetyl-CoA derived from β-oxidation of FFAs (Randle et al., 1963).

Correspondingly, the capacity for pyruvate oxidation was found to be depressed in several rodent models of diabetes. A number of studies have employed hyperpolarized-^13^C MRS to measure flux through PDH, the key enzyme linking glycolysis to the mitochondrial citric acid cycle (Schroeder et al., 2008; Le Page et al., 2015; Rohm et al., 2018). Flux through PDH was found to be depressed across multiple rodent models including the STZ rat model of T1DM, the low dose STZ + HFD-fed rat model of T2DM (Mansor et al., 2013) and βV59M mice (Rohm et al., 2018). One study in the STZ rat also reported that the degree of impairment of PDH flux correlated with severity of the diabetic phenotype (as determined by the extent of blood glucose elevation) (Schroeder et al., 2008), which implicates restricted pyruvate oxidation in the etiology of DbCM, although whether this is causal has yet to be determined.

There is, however, conflicting evidence from metabolomic studies regarding FA oxidation in the diabetic myocardium, with studies reporting both increased and decreased capacities. Three such studies utilized retrograde perfusion of *ex vivo* hearts and included ^13^C-labeled FA in the perfusate to determine FA oxidation by NMR through quantification of the incorporation of the ^13^C-label into the TCA cycle metabolite pool (Chatham and Forder, 1997; Chatham et al., 1999; Wang et al., 2005). The apparent discrepancies in findings may be a result of the model used and/or the pathology studied. FA oxidation was found to be decreased in the hearts of T1DM STZ rats (Chatham and Forder, 1997; Chatham et al., 1999), whilst FA oxidation was elevated in comparison with controls in the T2DM ZDF rat (Wang et al., 2005). Differences in metabolic features of the development of DbCM in T1DM compared with T2DM may underlie these apparent discrepancies, and these have been comprehensively reviewed elsewhere (Hölscher et al., 2016). Further differences between these two models may reflect the progressive nature of DbCM, with lower FA oxidation in the STZ rat perhaps reflecting a more severe form of DbCM (Chatham and Forder, 1997; Chatham et al., 1999) characterized by impairment of total oxidative capacity and loss of cardiac mitochondrial content, whilst increased FA oxidation in the ZDF rat (Wang et al., 2005) may reflect an earlier, adaptive stage of DbCM development. Moreover, in all these studies FA oxidation was investigated in hearts perfused *ex vivo*; a setting which may not fully capture the complexity of diabetic conditions *in vivo* (Larsen and Aasum, 2008).

Methodological limitations may also influence interpretation of results, however, since absolute rates of flux were not measured. The studies reporting depressed FA oxidation in the STZ rat instead reported relative rates of flux, and thus indicated substrate preference or metabolic flexibility. For instance, when perfused with both fatty acids and glucose, a lower percentage of substrate entering the citric acid cycle was derived from fat in the diabetic heart compared with controls (Chatham and Forder, 1997). Meanwhile the STZ hearts showed a reduced ability to alter FA oxidation in response to changing concentrations of substrate, with a 10-fold increase in exogenous palmitate only increasing FA oxidation by 30% in the diabetic heart, compared with a sevenfold increase in control rats (Chatham et al., 1999). Since absolute oxidation rates could not be measured, it is possible that these studies do not contradict the finding of increased FA oxidation in the diabetic heart, which was derived from measures of ATP production from different metabolic substrates (Wang et al., 2005). Instead, the results may reflect a higher overall metabolic rate in the diabetic heart, with the relative contribution of fat being reduced (Chatham and Forder, 1997) and the capacity to increase FA oxidation in response to increased substrate delivery limited (Chatham et al., 1999).

The conflicting evidence arising from these studies, highlights why metabolomic techniques should not be considered in isolation. FA oxidation, has been investigated by a number of other, non-metabolomic techniques, including PET (Herrero et al., 2006; van den Brom et al., 2009), radiolabeled-substrate oxidation measurements (How et al., 2006), and measurements of mitochondrial oxidative capacity by respirometry (Kuo et al., 1983; Vazquez et al., 2015). Such findings are generally in agreement that FA oxidation is increased in the diabetic heart, being seen in both animal models of T1DM (STZ mice, How et al., 2006) and T2DM [both the *db/db* mouse (How et al., 2006) and ZDF rat (van den Brom et al., 2009)], as well as women with T1DM (Herrero et al., 2006). It should be noted, however, that studies of FA oxidative capacity measured in isolated mitochondria do show some conflicting results, with a decreased palmitoyl carnitine-supported OXPHOS capacity reported in mitochondria from *db/db* mouse hearts (Kuo et al., 1983), but an increased capacity for palmitoyl-carnitine supported OXPHOS in subsarcolemmal mitochondria isolated from the hearts of STZ-rats (Vazquez et al., 2015). It is worth noting, however, that respirometry studies of mitochondrial capacity are carried out at saturating substrate and oxygen concentrations, and thus provide a robust measurement of respiratory capacity, but may not reflect that which is occurring *in vivo* (Lanza and Nair, 2009).

Application of metabolomic techniques to investigate general changes in the cardiac lipidome have provided further evidence for alterations to fatty acid oxidation in the diabetic myocardium, in the form of changes in levels of fatty acid or acylcarnitine species. In the STZ-induced diabetic rat heart, accumulation of long-chain (and 3-hydroxylated) acylcarnitine species has been seen (Su et al., 2005), suggesting a mismatch between delivery of fatty acid substrate delivery and oxidation rate in this model. This study also highlights the power of using metabolomic techniques for investigating changes in lipid metabolism, as although elevated levels of total acylcarnitine species have been associated with high rates of FA oxidation (Schooneman et al., 2013), the ability to distinguish different chain lengths has demonstrated that accumulation of long-chain acylcarnitine species specifically is often associated with β-oxidation dysfunction (Kler et al., 1991). Moreover, alterations in specific fatty acid species have also been observed: in the Akita mouse model of T1DM, long-chain fatty acid species accumulated in the myocardium (Basu et al., 2009), whilst in the βV59M mouse, relative levels of the shorter chain myristic acid (C14) and two unsaturated fatty acids (C16:1 and C18:1) were lower than in non-diabetic controls (Rohm et al., 2018).

**Table 4:** Changes in lipid metabolites observed to occur in the diabetic myocardium through metabolomic studies in animal models.

Model	Lipid metabolites profiled											Method	References
	FA	TAG	DAG	Ch	Cer	CL	PC	PE	PG	PI	PS		
βV59M mice	↓											GC–MS	Rohm et al., 2018
Akita mice	↑	↑	↑		↑							HPLC	Basu et al., 2009
*MHC-PPAR* mice + STZ		↑										ESI-MS	Finck et al., 2003
Mice + HFSD		↑										^1^H-MRS	Abdesselam et al., 2015
*ob/ob* mice + HFD			↑									LC–MS/MS	Wang et al., 2015
*Gpat1^−/−^* mice							↕	↕		↕	↕	GC–FID	Lewin et al., 2008
Mice + STZ		↑				↕						QToF MS	Han et al., 2007
Mice + STZ						↓			↓			MS/MS	Han et al., 2005
Rat + STZ		↑										NMR	O’Donnell et al., 2006
Rat + Alloxan		↑										TLC	Denton and Randle, 1967
Zucker Fatty Rat		↑										HPTLC	Coort et al., 2004
Rat + STZ				↑								GC	Matsui et al., 1997

*↓ decreased abundance in diabetic hearts, ↑ increased abundance in diabetic hearts, ↕ specific species increased and others decreased in abundance in diabetic hearts. HFD, high fat diet; Gpat1, glycerol-3-phosphate acyltransferase 1; MHC-PPAR, myosin heavy chain-peroxisome proliferator-activated receptor gene cassette; STZ, streptozotocin; HFSD, high-fat high-sucrose diet; FA, fatty acid; TAG, triacylglycerol; DAG, diacylglycerol; Ch, cholesterol; Cer, ceramide; CL, cardiolipin; PC, phosphatidylcholine; PE, phosphatidylethanolamine; PG, phosphatidylglycerol; PI, phosphatidylinositol; PS, phosphatidylserine; HPLC, high-performance liquid chromatography; GC, gas chromatography; LC, liquid chromatography; MS, mass spectrometry; FID, flame ionization detector; ESI, electronspray ionization; QToF, quadrupole time of flight; MRS, magnetic resonance spectroscopy; NMR, nuclear magnetic resonance; TLC, thin-layer chromatography; HPTLC, high-performance thin-layer chromatography.*

Taken together, these results suggest that glucose metabolism is suppressed in both T1DM and T2DM. Meanwhile, metabolomic studies generally support findings that fatty acid oxidation is elevated in the diabetic heart, although some inconsistencies do highlight the importance of not considering metabolomics in isolation.

### Other Alterations to Lipid Metabolism

Beyond FA oxidation, alterations to other pathways of lipid metabolism are evident in DbCM, and may play an important role in the progression of the pathology. Indeed, one proposed mechanism for the development of DbCM holds that despite an initial compensatory increase in FA oxidation capacity, lipid overload results in the accumulation of harmful lipid intermediates, a phenomenon known as lipotoxicity (Unger and Orci, 2000). This, in turn, has been suggested to result in cardiac mechanical dysfunction. Evidence from animal models to support such lipid accumulation and altered lipid metabolism in the diabetic heart is summarized in Table 4.

Lipotoxicity results from ectopic lipid accumulation, and many studies have demonstrated that the total TAG species pool is elevated in the diabetic myocardium of human T2DM cohorts (McGavock et al., 2007; Rijzewijk et al., 2008; Levelt et al., 2016a) and animal models (Denton and Randle, 1967; Finck et al., 2003; Coort et al., 2004; O’Donnell et al., 2006; Basu et al., 2009; Abdesselam et al., 2015). In animals, myocardial TAG accumulation seems to occur regardless of the model investigated or type of diabetes. For example, TAG accumulation has been observed in both mice and rats treated with STZ or alloxan (Denton and Randle, 1967; Finck et al., 2003; O’Donnell et al., 2006; Han et al., 2007), Akita mice (Basu et al., 2009), mice overexpressing peroxisome proliferator-activated receptor-alpha (PPARα) and treated with STZ (a severe T1DM phenotype) (Finck et al., 2003), mice fed a diabetogenic high-fat, high-sugar diet (Abdesselam et al., 2015) and the Zucker fatty rat (Coort et al., 2004). An increased myocardial TAG pool has also been associated with obesity in both human and rat studies, correlating with body mass index (Szczepaniak et al., 2003; Reingold et al., 2005; Kankaanpää et al., 2006; Utz et al., 2013), and since obesity is an important risk factor for the development of diabetes (NCD Risk Factor Collaboration (NCD-RisC), 2016), it is plausible that TAG accumulation is causative in the development of DbCM. This hypothesis is supported by a number of ^1^H-MRS studies coupled to MRI, which found that increased myocardial TAG concentration correlates with impaired diastolic function (Hammer et al., 2008a,b; Rijzewijk et al., 2008) or myocardial strain (Ng et al., 2010).

In apparent contrast, however, the administration of insulin therapy to treat T2DM was shown to acutely raise myocardial TAG concentrations with no apparent functional consequences (Jankovic et al., 2012). Meanwhile, the development of systemic insulin resistance in non-diabetic heart failure patients is associated with decreased rather than increased myocardial TAGs (Chokshi et al., 2012). Treatment with two anti-glycemic agents (pioglitazone and metformin) had no effect on myocardial TAG concentration in men with uncomplicated T2DM compared with those treated with placebo (van der Meer et al., 2009). Finally, in a population with metabolic syndrome, though not overt T2DM, myocardial TAG content was not independently associated with diastolic dysfunction (Nyman et al., 2013).

It is therefore possible that myocardial steatosis resulting from TAG accumulation is not the driving force behind the development of DbCM. Indeed, cellular lipotoxicity is also associated with accumulation of several other specific lipid species, including DAGs and ceramides. Several studies, again in both animal models and human diabetic cohorts, have demonstrated accumulation of DAGs and ceramides in the diabetic myocardium both alongside TAG accumulation (Basu et al., 2009), and in the absence of TAG accumulation (Chokshi et al., 2012; Wang et al., 2015). DAG accumulation occurs in Akita mice (Basu et al., 2009) and HFD-fed *ob/ob* mice (Wang et al., 2015). DAG accumulation has been shown to correlate with insulin resistance (Zhang et al., 2011), further implicating lipotoxic mechanisms in the etiology of DbCM. In studies of right atrial samples from healthy, obese and obese+T2DM patients, however, the total content of ceramides were found to be similar across all three groups, although the levels of individual ceramide species were not reported (Baranowski et al., 2010).

Increased FA uptake and oxidation in the diabetic heart may also increase mitochondrial production of reactive oxygen species (ROS) (Bugger and Abel, 2010), causing oxidative stress and accelerating the development of DbCM. Lipid peroxidation has been seen in the STZ rat heart, and this was preventable with insulin treatment (Jain and Levine, 1995), whilst oxidized lipids were elevated in the cardiac muscle of the STZ mouse (Towner et al., 2015). The latter study also utilized a 5,5-dimethyl-1-pyrroline-N-oxide (DMPO) probe to detect free radicals by NMR, and found that these increased in the diabetic heart with specific regional hotspots in the apical and upper left areas of the left ventricle (Towner et al., 2015). Furthermore, in a study that reported DAG accumulation in the myocardium of *ob/ob* mice fed a HFD, there were increased levels of 8-oxoguanine and an increased ratio of oxidized/reduced glutathione, indicative of oxidative stress (Wang et al., 2015), further demonstrating an association with myocardial lipotoxicity.

### Lipidomics: A Tool for Biomarker Discovery in DbCM

The development and progression of DbCM is associated with altered levels of specific lipid species in the myocardium, which has led to the suggestion that lipids might act as plasma-borne biomarkers for the condition. Shotgun lipidomic studies have often been used for this purpose (Han et al., 2005, 2007; Dong et al., 2017, 2018). PC, PE, and SM species are among the lipid classes identified as potential biomarkers for DbCM (Dong et al., 2017, 2018), with a number of species correlating with changes in cardiac function (Dong et al., 2017). Specifically, the species PC[22:6/18:2], PC[22:/18:1], PC[20:4/16:1], PE[20:4/18:2], and PE[20:4/16:0] were all found to be downregulated, and PC[20:2/18:2], PC[18:0/16:0], and PC[20:4/18:0] upregulated in the myocardium of a high-sucrose/fat diet +STZ rat model (Dong et al., 2017), with some perturbations in lipid profiles (e.g., PC[20:4/18:0]) being partially reversible upon treatment with the phytochemical berberine, the active ingredient of a traditional Chinese medicine used in the treatment of diabetes (Dong et al., 2018). Increased levels of PC and PE species in the sarcoplasmic reticulum membrane were also shown to occur in the diabetic myocardium, with decreased levels of species containing arachidonic acid (20:4) and increased levels of those containing docosahexaenoic acid (22:6) being detected (Kuwahara et al., 1997). In addition, changes in several plasma sphingolipid species were found to correlate with improved fasting insulin in patients with T2DM (Airhart et al., 2016).

Whilst the findings outlined above demonstrate that induction of diabetes is associated with changes in lipid metabolism, the reverse is seen in the *Gpat1^−/−^* mouse. This mouse was primarily developed as a model of altered cardiac FA metabolism, and in turn it develops some characteristics of diabetes (Lewin et al., 2008), highlighting a possible causal link between alterations in lipid metabolism and DbCM. The *Gpat1^−/−^* mouse is characterized by increased cardiac levels of PC, PE, PS, and PI species with increased arachidonic acid, although this was also accompanied by a decrease in 16:0-FA containing PC, PE, PS and PI, and increased PC and PE species containing 18:0 and 18:1 (Lewin et al., 2008).

Other proposed lipid biomarkers include the oxysterol species 7-β-hydroxycholesterol and 7-ketocholesterol (Matsui et al., 1997) and the relative levels of different cardiolipin species, with a redistribution of those containing 18:2-FAs to those containing 22:6-FAs detected in STZ-induced diabetic rodent models (Han et al., 2007), with the latter finding preceding TAG accumulation in the myocardium. A general decrease in the concentrations of mitochondrial cardiolipin species was found in the hearts of STZ mice using shotgun lipidomics (Han et al., 2005). This was likely bought about by reduced cardiolipin synthesis, since the metabolic precursor for cardiolipin, phosphatidylglycerol, was also found to be depleted (Han et al., 2005).

The studies outlined above have mainly examined cardiac tissue from animal models of DbCM and highlight the possibility that unique changes in lipid metabolism occur alongside the pathology. Changes in the plasma lipid profile have been observed in a lipidomic pilot study, where patients with T2DM fed a diet rich in medium chain FAs demonstrated decreased circulating levels of several sphingolipid, ceramide, and acylcarnitine species implicated in DbCM, whilst patients fed a diet rich in long chain FAs did not (Airhart et al., 2016). Whilst further studies are clearly needed, this highlights the potential of lipidomic approaches for the discovery of plasma-borne biomarkers for DbCM.

## A Role for Ketone Metabolism in the Diabetic Myocardium?

Oxidation of the ketone bodies, acetone, acetoacetate and β-hydroxybutyrate, represents a relatively small, but potentially important source of myocardial ATP. In a ^13^C-NMR study of the perfused healthy rat heart, the inclusion of labeled glucose, lactate, acetoacetate and long-chain FAs at physiological concentrations demonstrated that, whilst FA oxidation made the largest contribution to the cardiac acetyl-CoA pool, 23% of acetyl-CoA was derived from acetoacetate (Jeffrey et al., 1995). This suggests that, when ketone bodies are present, there is some preference for ketone oxidation to meet cardiac energy requirements in the healthy heart. Two studies utilizing metabolomic methods, reported that ketone oxidation was elevated in the (non-diabetic) failing heart of mice and humans (Aubert et al., 2016; Bedi et al., 2016), highlighting the importance of ketone metabolism in cardiac pathology.

In diabetic patients, however, ketosis is a serious clinical complication that requires immediate treatment to prevent life-threatening acidosis (Chong et al., 2017) and ketones are therefore usually considered to be detrimental in diabetes. Perhaps because of this, there are currently few published studies that have investigated ketone metabolism in the diabetic heart, although a small number do suggest that cardiac ketone metabolism is altered in diabetes. Through GC–MS analysis of arterial and coronary sinus blood, the myocardium of insulin-dependent diabetics was shown to extract more ketone bodies that that of healthy control patients (Avogaro et al., 1990). Cardiac levels of β-hydroxybutyrate were also found to become elevated over the progression of diabetes in an open-profile ^1^H-NMR study of the STZ rat (Zhao et al., 2018). Conversely, administration of empagliflozin, an SGLT2 inhibitor, in a T2DM patient cohort to reduce hyperglycemia, increased cardiac levels of β-hydroxybutyrate (Ferrannini et al., 2016). Whilst these studies indicate alterations in ketone metabolism in DbCM, whether this represents an adaptation of the heart to diabetes or a causative factor in the pathogenesis of DbCM needs further investigation.

## Amino Acid Metabolism and Diabetic Cardiomyopathy

Recent interest has emerged in BCAA metabolism in the context of T2DM pathogenesis, with many studies suggesting that leucine, isoleucine, and valine are involved in the development of insulin resistance (Bloomgarden, 2018). In the βV59M mouse, the accumulation of metabolites was investigated by GC–MS and all three BCAAs were found to be elevated in comparison with non-diabetic controls (Rohm et al., 2018). Meanwhile in the STZ rat heart, ^1^H-NMR spectroscopy showed that valine, leucine, and isoleucine all became elevated during the progression of diabetes (Zhao et al., 2018). Furthermore, BCAAs were found to be released by the T1DM human heart, but not by healthy hearts (Avogaro et al., 1990), providing further evidence for elevation of BCAA production or depressed catabolism in the diabetic myocardium.

It is noteworthy, however, that other aspects of amino acid metabolism may be altered in the diabetic heart. Metabolic profiling of a diabetic rat model which expresses human pancreatic amylin (HIP-model) highlighted several amino acid species (including phenylalanine, tyrosine, glycine, serine, and lysine) the concentrations of which were altered in the diabetic hearts (Ilaiwy et al., 2016). Although the HIP-model is a very specific diabetic phenotype, where diabetes is induced over time through increased proteotoxicity in pancreatic β-cells, the authors found that the metabolome of their model demonstrated a similar fall in glycine levels and increase in lysine compared with strain-matched T2DM rats in a untargeted metabolomic study (Ilaiwy et al., 2016), indicating that altered amino acid metabolism may be a common metabolic signature of the diabetic heart.

## Discussion and Future Perspectives

Metabolomic techniques are proving to be valuable in helping to unravel the metabolic disturbances of DbCM. Open profiling methods may lead to the identification of putative biomarkers in animal models of DbCM, whilst targeted approaches have highlighted alterations in lipid, ketone, and amino acid pathways.

Despite this, unanswered questions remain regarding metabolism in the diabetic heart, which could be addressed in future studies using metabolomic techniques. Firstly, the time course of metabolic derailment in DbCM is unclear. Open-profiling experiments, coupled with longitudinal study designs, could be useful in elucidating this timeline. Lack of a conclusive time course also means that the causal relationship between changes in metabolism and cardiac dysfunction has not been shown; although metabolic derailment is often considered to be causal in the progression of the diabetic myocardium toward overt failure, changes in metabolism may in turn arise as a consequence of mechanical dysfunction. However, if aspects of metabolic remodeling are indeed causal in the development of DbCM, then mechanistic detail needs to be elucidated. Here, more targeted metabolomic techniques will be useful. By monitoring changes in key metabolic pathways, the stage at which alterations to metabolism occur may become apparent. This strategy might highlight enzymes or subcellular compartments (e.g., mitochondria versus cytoplasm) affected by DbCM to suggest mechanisms underpinning dysfunction. Meanwhile, the results of studies demonstrating alterations in cardiac metabolism in diabetes need to be placed in context, and here complementary techniques are useful in indicating how changes in metabolite concentrations or flux correspond to alterations in, e.g., mitochondrial respiratory capacity (Ashmore et al., 2014; Wang et al., 2015) or cardiac function *in vivo* (Le Page et al., 2015) or *ex vivo* (Dodd et al., 2018). Further work is needed to determine whether elevated myocardial ketone bodies are beneficial or detrimental to the diabetic heart. Finally, it is important to remember that diabetes is a systemic condition, and therefore changes in metabolism in organs other than the heart may also play a role in the progression of dysfunction in the diabetic heart. Accordingly, the interactions between the heart and other organs require further investigation.

**FIGURE 1:**
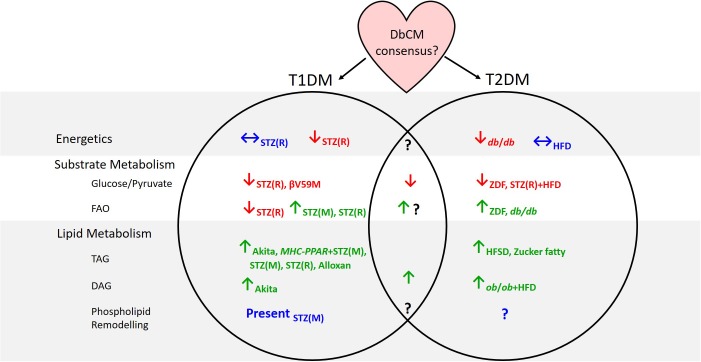
Summary of evidence from animal models concerning cardiac metabolic alterations in type-1 and type-2 diabetes. Subscripts denote the models in which investigations have been carried out. T1DM, type-1 diabetes; T2DM, type-2 diabetes; DbCM, diabetic cardiomyopathy; FAO, fatty acid oxidation; TAG, triacylglycerol; DAG, diacylglycerol; STZ(R), streptozotocin-rat model; STZ(M), streptozotocin-mouse model; MHC-PPAR, myosin heavy chain-peroxisome proliferator-activated receptor gene cassette; HFD, high fat diet; ZDF, Zucker diabetic fatty; HFSD, high-fat high-sucrose diet.

Some inconsistent findings surrounding metabolism in the diabetic myocardium may have arisen through differences in animal models used, or the type of diabetes studied. Indeed, whilst metabolic derangements are apparent in the hearts of rodent models of both T1DM and T2DM, the clinical presentation of DbCM in T1DM patients has been considered more controversial than in T2DM cohorts (Hölscher et al., 2016). Metabolomic studies have shown that many similarities exist between T1DM and T2DM hearts, with depressed glucose and pyruvate oxidation and accumulation of TAGs and DAGs seen regardless of the model studied (Figure 1). Discrepancies have been reported between studies of cardiac energetics in different T2DM models, and this might be explained by the severity of the model or the timepoint studied, since DbCM is a progressive condition. Disagreement on the pattern of changes in fatty acid oxidation between models of T1DM and T2DM, may reflect the nature of the T1DM model. Whilst STZ treatment is the most commonly used method of generating T1DM, it is known to have effects beyond the ablation of pancreatic β-cells, for example, causing cytotoxicity in liver and kidney owing to the high density of GLUT2 in these organs (Lenzen, 2008). GLUT2 is not expressed in heart, and STZ is therefore unlikely to accumulate in cardiac tissue (Schnedl et al., 1994), but may nevertheless exert effects on cardiac metabolism and mitochondrial function through mechanisms beyond insulin depletion. Future studies that investigate fatty acid oxidation via metabolomic methods in other models of T1DM, such as the NOD mouse or the surgical rat pancreatectomy model (Thisted et al., 2018) would be therefore informative.

A significant disadvantage of metabolomic techniques is that findings typically reflect metabolite levels at a single time point, whereas *in vivo* metabolite concentrations are rarely static (although they may be at steady state) and metabolites constantly flux through a variety of metabolic pathways. Some techniques have tried to overcome this limitation including hyperpolarized-^13^C MRS studies measuring, for example, PDH flux (Schroeder et al., 2008; Le Page et al., 2015; Rohm et al., 2018), and stable isotope tracing experiments, designed to observe which metabolites accumulate following administration of a specific labeled substrate (e.g., Chatham and Forder, 1993, 1997; Chatham et al., 1999; Chatham and Seymour, 2002; Wang et al., 2005; O’Donnell et al., 2006). Nevertheless, metabolomic techniques should be combined with other techniques to provide a comprehensive overview of metabolism in the diabetic heart. This is especially evident when considering FA oxidation in the diabetic heart; metabolomic approaches suggest conflicting evidence regarding whether FA oxidation is elevated in DbCM, whilst non-metabolomic methods are more conclusive (Figure 1). It is therefore imperative that metabolomics are not considered in isolation.

One such technique, that has been utilized widely in human studies (e.g., Monti et al., 2004; Peterson et al., 2004; Herrero et al., 2006; Bucci et al., 2012), and some animal models (e.g., Welch et al., 2006; Shoghi et al., 2008; van den Brom et al., 2009), is PET. Whilst often used to aid disease diagnosis, especially in the diagnosis of cancer metastasis, PET can be used to investigate alterations to metabolism. Substrates, labeled with a radioactive tracer such as ^18^F, ^11^C, ^13^N and ^15^O, are taken up by metabolically-active tissues including the heart, and radioactive decay of the tracer can then be measured, indicating uptake and flux of the tracer in tissues of interest. Although expensive, owing to the cost of producing the tracers and instrumentation, metabolic and cardiovascular parameters such as myocardial blood flow, myocardial oxygen consumption, and substrate specific uptake, utilization, and oxidation (Peterson et al., 2004) can be measured *in vivo*, making PET a useful, non-invasive tool for investigating metabolism in DbCM. Combining PET with metabolomics (e.g., Rijzewijk et al., 2009; Bucci et al., 2012; Abdurrachim et al., 2017) therefore has the potential to reveal *in vivo* changes in flux of targeted metabolites and to identify novel alterations in metabolic pathways in an unbiased manner.

Finally, novel applications of metabolomic techniques may also help to elucidate metabolic changes in the diabetic myocardium. MSI is an application of MS that enables the distribution of tissue metabolites to be analyzed *in situ* (Hall et al., 2017). Unlike traditional MS, MSI does not damage the integrity of the tissue of interest, as metabolites are directly ionized from the surface of the tissue, allowing a comparison to be drawn between metabolite distribution and underlying tissue histology (Addie et al., 2015). This technique has been used, for example, to demonstrate changes in the hepatic zonation of lipids in non-alcoholic fatty liver disease (Hall et al., 2017) and alterations to phospholipid distribution across an infarction in the rat heart (Menger et al., 2012). It could therefore be beneficial to apply MSI to the diabetic myocardium to assess spatial changes across the heart over the progression of DbCM and relate them to contractile dysfunction or local energetic profiles in order to fully elucidate the mechanism of metabolic pathology.

## Conclusion

Metabolomic studies have helped to elucidate metabolic derangements that occur in the diabetic heart. Glycolysis and glucose oxidation are depressed in DbCM and it is highly likely that this is accompanied by energetic impairment. Open-profiling lipidomic studies have demonstrated that that there are specific classes of lipid, and individual species within these classes, that are distinctly altered in the diabetic myocardium and some of these species might serve as biomarkers for DbCM, particularly if they relate to alterations in circulating lipid species. There are, however, questions remaining concerning the physiological significance of lipid remodeling and the mechanistic role this might play in the pathology. For instance, whilst it has been postulated that lipotoxicity may be a driving force behind functional impairment, whether changes in individual lipid species are causal in the development and/or progression of DbCM, or a simply consequence of the condition needs further investigation.

## Author Contributions

All authors listed have made a substantial, direct and intellectual contribution to the work, and approved it for publication.

## Conflict of Interest Statement

The authors declare that the research was conducted in the absence of any commercial or financial relationships that could be construed as a potential conflict of interest.

## Abbreviations

ADP: adenosine diphosphate
ATP: adenosine triphosphate
BCAA: branched-chain amino acid
CAD: coronary artery disease
DAG: diacylglycerol
DbCM: diabetic cardiomyopathy
ESI: electronspray ionization
FA: fatty acid
FFA: free fatty acid
FID: flame ionization detector
GC: gas chromatography
HFD: high fat diet
HFSD: high fat, high sucrose diet
HPLC: high-performance liquid chromatography
HPTLC: high-performance thin-layer chromatography
LC: liquid chromatography
MRI: magnetic resonance imaging
MRS: magnetic resonance spectroscopy
MS: mass spectrometry
MSI: mass spectrometry imaging
NMR: nuclear magnetic resonance
OXPHOS: oxidative phosphorylation
PC: phosphatidylcholine
PCr: phosphocreatine
PDH: pyruvate dehydrogenase
PE: phosphatidylethanolamine
PET: positron emission tomography
PI: phosphatidylinositol
PS: phosphatidylserine
QToF: quadrupole, time of flight
SM: sphingomyelin
STZ: streptozotocin
T1DM: type-1 diabetes mellitus
T2DM: type-2 diabetes mellitus
TAC: thoracic aortic constriction
TAG: triacylglycerol
TCA: tricarboxylic acid
TCD: thermal conductivity detector
TLC: thin-layer chromatography
ZDF: Zucker diabetic fatty rat.
